# Design and Research of Wireless Passive High-Temperature Sensor Based on SIW Resonance

**DOI:** 10.3390/mi13071035

**Published:** 2022-06-29

**Authors:** Fujia Xu, Shujing Su, Lili Zhang, Ting Ren

**Affiliations:** State Key Laboratory of Dynamic Measurement Technology, School of Instrument and Electronics, North University of China, No.3 Xueyuan Road, Taiyuan 030051, China; x1186159946@163.com (F.X.); lilizhang@llu.edu.cn (L.Z.); ritty_a@126.com (T.R.)

**Keywords:** substrate integrated waveguide, resonant cavity, silicon carbide, high temperature sensor, wireless passive

## Abstract

The temperature of advanced components in aviation and aerospace fields is difficult to obtain timely. In this study, we aimed to investigate microwave backscattering technology combined with the theory of substrate integrated waveguide and resonant cavity to design a wireless passive temperature sensor and explore its potential in this field. We employed silicon carbide and aluminum ceramic as the substrate to make sensors. The interrogation antenna was designed to test the sensor, which could completely cover the working frequency of the sensor and had good radiation characteristics. Based on the test results, the silicon carbide sensor was capable of bearing a temperature limit of about 1000 °C compared to the alumina sensor. From 25 °C to 500 °C, its sensitivity was 73.68 kHz/°C. Furthermore, the sensitivity was 440 kHz/°C in the range of 501 °C to 1000 °C. Moreover, we observed the surface of this sensor by using the scanning electron microscope, and the results showed that the damage to the sensor surface film structure caused by long-term high temperature is the major reason for the failure of the sensor. In conclusion, the performance of the silicon carbide sensor is superior to the alumina sensor.

## 1. Introduction

Nowadays, it is vital to monitor timely the temperature of aero-engines and advanced aviation components in the fields of aviation and aerospace [1]. However, wired active devices have problems, for example, easy damage to lines, structural instability in high-temperature environments, and power supplies with other components that cannot bear high temperature. For example, thermocouples and thermal resistors are contact temperature measurement techniques, which have many wirings and are complicated. The technology is susceptible to electromagnetic interference and has poor stability. Thereby, it is extremely urgent to develop wireless passive temperature measurement systems [2,3,4].

The ultra-high temperature sensor should be based on a wireless passive mechanism, ensuring long-term and stable work in harsh environments such as high temperature, high spin, and multi-metal. In addition, the substrate of the thermometric sensor should benefit from the robust sensing material, making the sensor strong, stable, and not easily deformed [5,6].

The wireless transmission is able to successfully transfer by the surface acoustic wave (SAW), Inductive Capacitive (LC), and Microwave Backscatter Temperature Sensors, avoiding cable packaging issues. Weng et al. used SAW technology to design a temperature sensor, which can work stably between 20 and 800 °C and has good repeatability [7]. Boldeiu et al. designed a temperature sensor based on GaN/SiC and GaN/sapphire SAW, which permit wide range, accurate temperature determinations. The sensor can work stably at −266–500 °C [8].

Milan et al. fabricated an LC temperature sensor consisting of a spiral inductor and an interdigital capacitor in parallel [9]. The LC sensor proposed by Ren et al. could simultaneously measure the temperature and humidity of the environment [10]. Another LC resonator fabricated by Tan et al. was reported to measure temperatures up to 700 °C [11]. However, the SAW sensors have disadvantages with unstable base material at high temperatures, and the test signal is easily disturbed by the environment [12,13]. Besides, low-quality factor, short measurement distance, and inability to attach to metal surfaces are the defects of the LC sensors, which make them not suitable for measuring temperature in harsh environments [14,15]. Recent studies reported that microwave backscattering technology has the characteristics of long transmission distance, strong anti-interference ability, high transmission quality factor, and high operating frequency (several GHz to tens of GHz) [16], which enables the sensors to be stable and long-term in harsh work environments. Hence, we exploited the wireless passive temperature sensor based on microwave backscattering technology in this paper.

The substrate-integrated waveguide (SIW) temperature sensor is a new type of waveguide structure. Its upper and lower layers of the substrate are sputtered with metal and metalized through holes on both sides that are connected to the upper and lower layers of metal to ensure the wave propagates along the substrate without scattering in other directions. The SIW has the merits of small size, compact structure, efficient transmission of millimeter-wave frequency bands, and easy integration with other devices. Furthermore, the SIW temperature sensor holds the characteristics of wireless passive and keeps stably working in harsh environments [17,18].

In this paper, we designed a novel wireless passive temperature sensor by using two ceramic materials as substrates. An inquiry antenna was designed to excite the sensor, and the sensor and the antenna were fabricated by screen printing and high-temperature sintering process together. Hereafter, the performance of the sensor was tested, and the sensor was microscopically observed by SEM to explore and analyze its damage mechanism as well as practicability of the sensor.

## 2. Temperature Measurement Principle

The system designed in this paper consists of four sections: Sensor, inquiry antenna, vector network analyzer, and data processing equipment. In the actual temperature measurement, the sensor was attached to the object, and the inquiry antenna was placed directly above the sensor and kept a certain distance from the sensor. The inquiry antenna was coupled with the sensor through electromagnetic waves. Other devices except the two pieces of equipment were in a relatively mild environment and had little impact by the environment.

**Figure 1:**
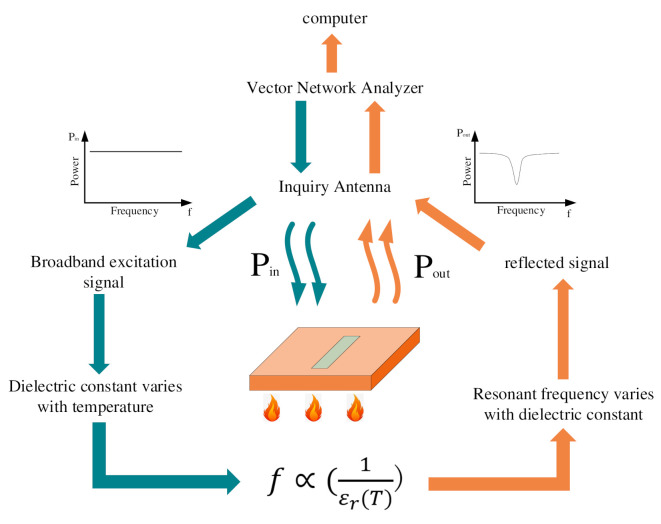
The working principle of the temperature sensing system.

The working principle of the test system is shown in Figure 1. The whole temperature measurement process is as follows: The vector network analyzer can send out a signal, including the resonant frequency of the sensor, and motivate the sensor through the interrogation antenna. At the resonant frequency, the energy of the signal will be dissipated by resonance, and the rest of the signal will be reflected to the inquiry antenna. The inquiry antenna delivers the signal to the vector network analyzer through the transmission line, subsequently, the resonant frequency and return loss S11 of the sensor can be obtained. 

When the temperature of the measured object increases, the dielectric constant of the dielectric material inside the sensor rises, leading to the resonant frequency shifts. The sensor feeds back the shift information to the vector network analyzer, and then the data is transmitted to the data device for the purpose of temperature measurement.

The principle of microwave resonant cavity is required to design the sensor. A microwave resonant cavity is a closed or nearly closed metal cavity, the interior of which is an empty cavity or filled with other dielectric materials. Microwaves are reflected back and forth in the cavity and form standing waves at the resonant frequency [19]. If a slot antenna is integrated into the resonator, the inquiry antenna can excite it and receive echoes. The resonance wavelength of the resonant cavity is calculated as Equation (1)

(1) $$\lambda_{0mnp}=\frac{2}{\sqrt{{\left(\frac{m}{a}\right)}^{2}+{\left(\frac{n}{b}\right)}^{2}+{\left(\frac{p}{l}\right)}^{2}}}$$

where *a*, *b*, *l* are the width, height, and length of the resonator, respectively. *m*, *n*, and *p* are positive integers, representing the number of standing waves distributed along *a*, *b*, and *l*, respectively. In practice, the permeability and permittivity of the filling medium in the cavity should be considered, so the corresponding resonance frequency is calculated as Equation (2)

(2) $$f_{0mnp}=\frac{v}{\lambda_{0mnp}}=\frac{c}{2\sqrt{\mu \varepsilon }}\sqrt{{\left(\frac{m}{a}\right)}^{2}+{\left(\frac{n}{b}\right)}^{2}+{\left(\frac{p}{l}\right)}^{2}}$$

In the Equation, *v* is the wave speed in the medium, *μ* and *ε* are the permeability and permittivity of the medium, respectively. In the rectangular resonator, there can be numerous H-type and E-type oscillation modes, the main oscillation mode is TE101, then the corresponding *λ*_0_ is shown in Equation (3)

(3) $$\lambda_{0}=\frac{2al}{\sqrt{a^{2}+l^{2}}}$$

The sensor is designed according to SIW principle and slot antenna theory. Substrate Integrated Waveguide (SIW) is a waveguide structure constructed by the upper and lower metalized plates of the dielectric substrate and the two rows of parallel metalized through-hole arrays on the sidewalls. W is the width of the SIW, and its relationship with the width of the resonant cavity is shown in Equation (4) [20].

(4) $$a=W-1.08\frac{4r^{2}}{p}+0.1\frac{4r^{2}}{W}$$

In the Equation, *r* is the radius of the metal through hole and *p* is the hole spacing. 

According to the above theory, the resonant frequency of the sensor is affected by the dielectric constant of the filling medium. The dielectric constant of the substrate material increases with the temperature rising, which leads to the resonant frequency of the sensor decrease. Hence, if the change of the resonant frequency of the sensor can be detected, the temperature can also be measured indirectly [21].

For this sensor, an interrogation antenna can be designed to excite it and receive the reflected signal. By processing the transmitted and received signals, the S11 (return loss) of the sensor can be obtained, and its resonant frequency can be determined, which makes the change of the resonant frequency of the sensor be monitored in real time to achieve the above temperature measurement purpose.

The inquiry antenna plays a central role, the structure of which is simple and has the ability of long-term stable operation in harsh environments. Coplanar waveguides have the features of small size, excellent design feasibility, no drilling required as well as coplanar floor and signal strips on the same plane, etc. Based on these great advantages, coplanar waveguides possess a significant potential in high frequencies microwaves [22,23]. To sum up, the coplanar waveguide is chosen as the antenna structure.

## 3. Design and Simulation Optimization

The resonant frequency of the preliminarily designed sensor is 9–12 GHz. According to the above theoretical calculations, the sensor corresponding to this frequency band is small in size, easy to attach and integrate, and this frequency band belongs to the X-band, which has the advantages of far detection and wide range [24,25]. Since the field distribution of the SIW is the same as that of the rectangular waveguide, the design of the slot antenna should refer to the field distribution of the rectangular waveguide and can radiate electromagnetic waves with maximum efficiency. 

Slot antennas cannot be located in the center of the SIW where the slot antennas cannot effectively radiate electromagnetic waves according to the electromagnetic field distribution. Increasing the number of slots will improve the electromagnetic radiation efficiency, but at the same time, the risk of damaging the SIW structure also will increase. 

**Figure 2:**
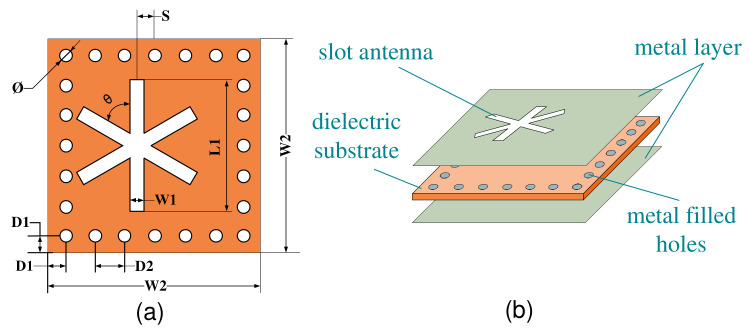
(**a**) Sensor plane structure, (**b**) sensor hierarchy structure.

The “∗” character structure was better, and the sensor structure was shown in Figure 2a. Figure 2b displays the hierarchical structure of the sensor. The middle layer was a ceramic substrate, and the upper and lower layers were metal layers. The through holes were filled with metal to connect the upper and lower metal layers, and the upper metal layer integrated a slot antenna. 

The width of the sensor was calculated according to the above Equation. As a tiny sensor, its length can be equal to its width, and a square sensor can be designed. The sensor should be easy to attach, so the thickness is set as H1 = 1 mm. We then model, simulate, and analyze the sensor in HFSS software. The main parameters affecting the resonant frequency in this structure are the distance S from the center of the slot antenna to the center of the rectangle, the width W1 and the side length L1, which are simulated and optimized, respectively. 

**Figure 3:**
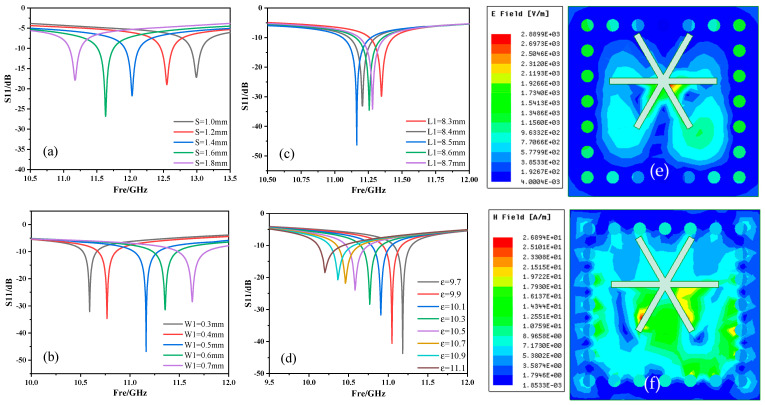
(**a**–**c**) The simulation results of the sensor structure parameters S, W1 and L1, (**d**) the simulation result of the sensor resonant frequency changing with the dielectric constant, (**e**,**f**) the electric and magnetic field distribution diagrams of the sensor after the simulation was completed.

The optimization result is shown in Figure 3. In the application process of the temperature measurement system, only the resonance point with a larger resonance depth can be selected for analysis to obtain higher sensitivity. Thereby, the parameter values are determined as S = 1.6 mm, W1 = 0.5 mm, and L1 = 8.5 mm.

According to the principle of temperature measurement, we obviously observed that the increase in temperature leads to an increase in the dielectric constant of ceramics. We changed the dielectric constant in the HFSS software to simulate the temperature change to obtain the resonance frequency. The simulation results show that with the increase of the dielectric constant, the resonant frequency gradually decreased with S11 gradually increasing. The resonant frequency shifts from 11.183 GHz to 10.188 GHz, which is shifted by 0.995 GHz, and S11 increases from −45.710 dB to −20.595 dB with an increase of 25.115 dB.

As shown in Figure 3e,f, the electric field and magnetic field of the sensor were distributed in different positions. The electric field was mostly concentrated in the center of the slot antenna, and the concentrated distribution of the electric field was conducive to the transmission of the signal. The magnetic field was mostly concentrated around the slot antenna, indicating that the slot antenna has good performance. The structural parameters of the sensor are shown in Table 1.

**Figure 4:**
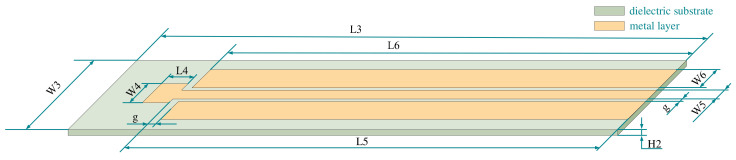
Interrogation antenna plane structure.

Next, the inquiry antenna design was carried out. The coplanar waveguide structure was used to design the antenna. The feeder was located in the center, with a large plane wrapping. The gap between the feeder and the ground plane was 0.5 mm. According to the 50 Ω transmission line theory and the calculation Equation of coplanar waveguide impedance, the width of the feeder was 1.43 mm. The use of a larger plane package, to a certain extent, improved the compactness and the gain and directivity of the antenna, maintaining the good performance of the antenna [26]. The structure of the designed antenna is shown in Figure 4.

**Figure 5:**
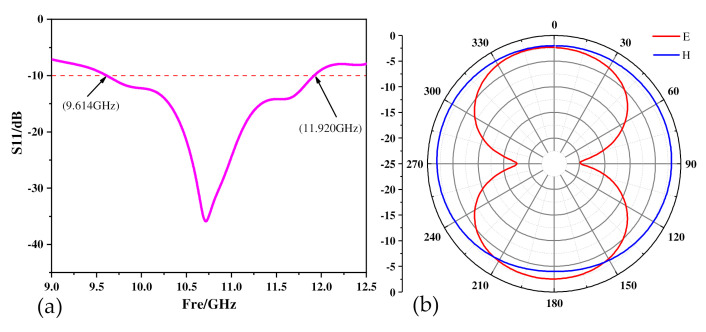
(**a**) Antenna working frequency band, (**b**) antenna pattern.

The working frequency of the interrogation antenna should completely cover the resonant frequency of the sensor and ensure that it can cover the range of frequency offset. The simulation results of the antenna in the HFSS software are shown in Figure 5. The operating frequency of the antenna is 9.614 GHz–11.920 GHz. The parameters of the antenna are shown in Table 2. 

Figure 5b shows the antenna patterns. The picture shows that the E surface is nearly symmetrical up and down, and the H surface is a hologram which can transmit and receive signals in all directions. The above shows that the antenna has good radiation characteristics.

## 4. Production Method

In this paper, silicon carbide ceramics and alumina ceramics were chosen as the two substrate materials. The dielectric constant of alumina ceramics is 9.8 and has excellent properties such as high strength, high hardness, high insulation resistance, high temperature resistance, corrosion resistance and wear resistance, which has been deeply studied in the field of wireless passive temperature sensors [27]. Silicon carbide material is a new type of ceramic material, and its dielectric constant is 9.7. The high bond energy of Si-C bond keeps silicon carbide a very inert material, making it difficult to be oxidized, as it resists chemical corrosion and radiation damage. Due to these advantages of wide band gap, high thermal conductivity, high mechanical strength, low thermal expansion coefficient, and strong radiation resistance, it has been widely used in sensors and power electronic devices working in extreme environments such as high temperature, high frequency, and pressure [28,29].

After determining the dimensional parameters of the sensor and inquiry antenna, they were manufactured by using laser cutting, drilling, and screen-printing processes. The upper and lower metal coatings and holes of the sensor were filled with 9633-G silver paste, which could afford a high temperature of 1000 °C and have relatively stable material properties in a high-temperature environment.

**Figure 6:**
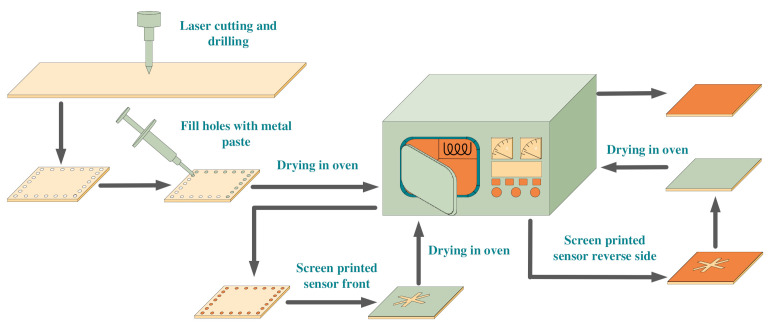
The production process of the sensor.

First, the holes were filled, then, the screen-printing craft was employed to fill the gap structure and the whole surface of the sensor. During the process, each step was required to be baked at 100 °C for 10 min to make it initially formed. Finally, it was put into a muffle furnace for high-temperature sintering at 1000 °C for 10 min, then, the power was turned off. The sensor was naturally cooled with the muffle furnace. High-temperature sintering at 1000 °C was able to remove the organic solvent and binder in the silver paste. After sintering, the silver particles would be rearranged on the ceramic sheet to form a dense silver metal film. The entire operation process was shown in Figure 6.

**Figure 7:**
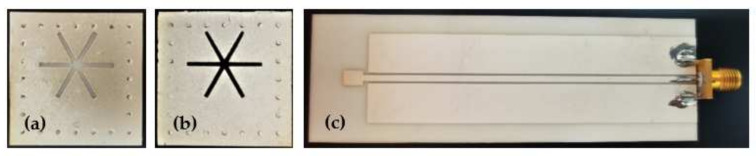
(**a**) Al_2_O_3_ ceramic sensor, (**b**) SiC ceramic sensor, (**c**) the inquiry antenna.

The alumina ceramic was used as the base of the antenna, and the fabrication method was similar to that of the sensor. The antenna substrate was processed by laser cutting, and then the coplanar waveguide structure was printed on the upper surface of the substrate by screen printing. Finally, it was placed in a muffle furnace for sintering at 1000 °C for 10 min, and slowly cooled to room temperature. After the antenna was fabricated, the tail end of the antenna was welded to the SMA head to prepare for subsequent testing. The final prepared sensor and antenna are shown in Figure 7 below.

## 5. Analysis of Testing Results

In the testing phase, a high-temperature test platform was first set up, which included five parts: The sensor, inquiry antenna, muffle furnace, vector network analyzer, and computer. The sensor was located in the muffle furnace, and the antenna was in front of the sensor with the distance 10 mm from the sensor. According to the simulation results during the actual test, we have continuously adjusted the distance between the sensor and the antenna. When the distance is 10 mm, the antenna’s transceiver effect is the best. This distance could keep balance of the distance and sensor performance to ensure that the antenna transceiver efficiency was capable of being maximized in order to get better testing results. 

**Figure 8:**
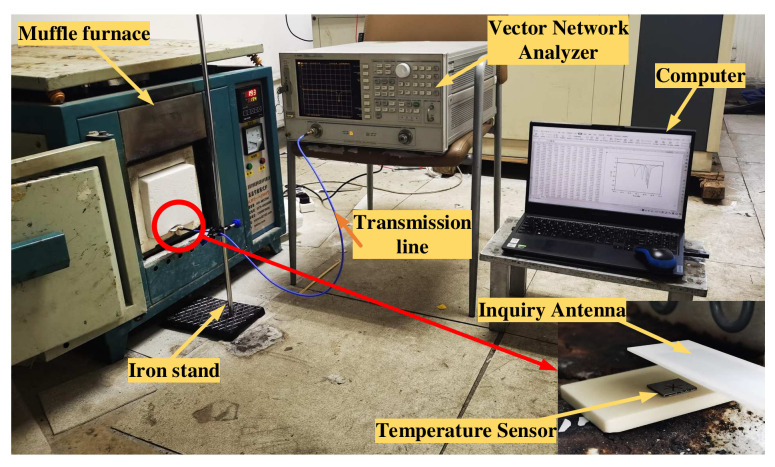
Test platform.

Half of the inquiry antenna was inside the furnace, and the other half was outside the furnace. The cold end of the antenna was separated by mullite to prevent the huge temperature difference from damaging the antenna and the equipment in the furnace. The cold end of the antenna was connected to the vector network analyzer through a transmission line. There was a temperature display device with a temperature control device in the muffle furnace to monitor the temperature timely in the furnace and control the heating rate and the holding time. The inner volume of the muffle furnace is 20 × 20 × 15 cm^3^ and the sensor is located near the thermocouple, so it can be considered that the measured temperature is the real-time temperature in the muffle furnace. The test platform is shown in Figure 8. 

**Figure 9:**
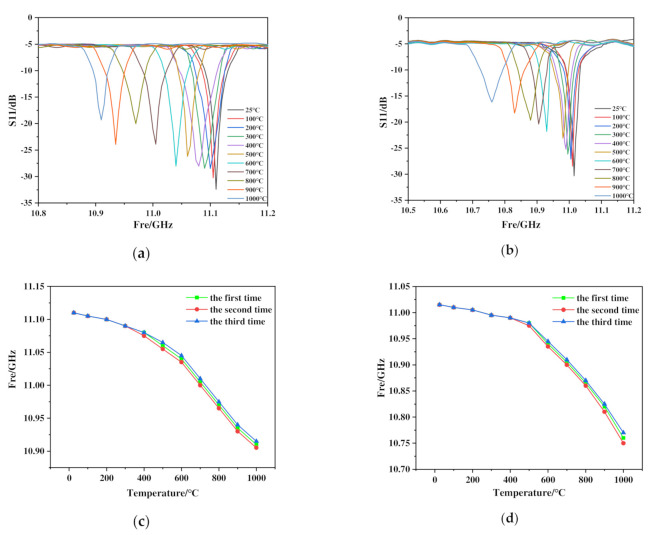
(**a**,**c**) The test results and repeated experiment results of Al_2_O_3_ ceramic sensor, (**b**,**d**) the test results and repeated experimental results of SiC ceramic sensor.

The two sensors were tested and analyzed, respectively, when the test platform was put up. According to the manual of the muffle furnace and the instructions provided by the ceramic processing manufacturer, The heating steps were as follows: First, the temperature was raised from 25 °C to 100 °C in 15 min, then, the temperature was raised linearly from 100 °C to 1000 °C in 90 min, and finally, the temperature was kept at 1000 °C for 10 min. During this period, data were recorded at room temperature firstly and kept noting every 100 °C thereafter. We turned off the power and let it cool down naturally when the whole test was completed. The testing results are shown in Figure 9.

As can be seen from Figure 9a,b, both sensors start to have a wide range of offset after 500 °C, which is somewhat deviated from the simulation results. The main reason is that the dielectric constant changes uniformly in the simulation, but in the actual test results, in order to more accurately analyze the linear characteristics of the sensor, it is divided into a low temperature range (25~500 °C) and a high temperature range (500~1000 °C) for analysis. In the low temperature range, the resonant frequency of Al_2_O_3_ ceramic sensor was 11.11 GHz, while the resonant frequency of SiC ceramic sensor was 11.015 GHz at room temperature 25 °C, which was close to the simulation results. When the temperature rising to 500 °C, the resonant frequency of Al_2_O_3_ ceramic sensor was 11.06 GHz and the sensitivity was 105.26 kHz/°C, however, the resonant frequency of SiC ceramic sensor was 10.98 GHz and the sensitivity of which was 73.68 kHz/°C. The above results demonstrate that the performance of Al_2_O_3_ ceramic sensor was better than that of SiC ceramic sensor in the low temperature range.

Strikingly, when the test temperature reached 1000 °C, the resonant frequencies of Al_2_O_3_ ceramic sensor and SiC ceramic sensor were 10.91 and 10.76 GHz, respectively, and the sensitivity of Al_2_O_3_ ceramic sensor was 300 kHz/°C, lower than that of SiC ceramic sensor which was 440 kHz/°C. The S11 parameter of SiC ceramic sensor increased regularly and gradually, with a total increase of 14.132 dB. Based on these results, the performance of SiC ceramic sensor was better than that of Al_2_O_3_ ceramic sensor in the high temperature range. As the test temperature was in the high temperature range in aerospace fields, the SiC ceramic sensor was preferred.

To verify the performance of SiC ceramic sensor, three independent repeated tests were conducted on the sensors under the original testing conditions. The testing results are shown in Figure 9c,d. There were some deviations in the testing results, which were caused by changes in the surface structure of the sensor on the microscopic level and the influence of environmental factors. However, in general, the frequency difference coincidence of SiC ceramic sensor was relatively high, which proved that the performance of SiC ceramic sensor was better. Therefore, we further confirmed that SiC ceramic sensor performs better than Al_2_O_3_ ceramic sensor in the high temperature range.

The temperature sensor proposed in this paper and other studies on similar sensors are illustrated in Table 3. Compared with the sensors in other published articles, the sensor studied in this paper had a smaller size and higher sensitivity, which is of great significance in wireless remote sensing, aerospace, and other fields.

**Figure 10:**
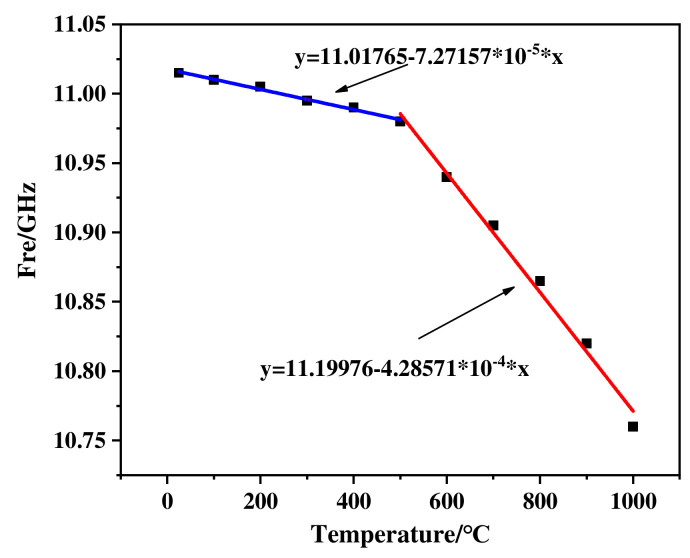
Linear fitting result of SiC ceramic sensor.

To accurately analyze the sensitivity of SiC ceramic sensor, piecewise linear fitting was performed on the resonant frequencies at different temperatures (Figure 10). This result indicates that the sensitivity of SiC ceramic sensor is better in the high temperature range. The reason for that was that the activity of the dielectric molecules inside the substrate increases with the external temperature rising, which leads to an accelerated change trend of the dielectric constant of the substrate. When the molecular activity increases to a critical level, the dielectric constant of the substrate, which is externally manifested, is significant, resulting in the appearance of an inflection point in the resonant frequency. The fitting curve Equations of SiC ceramic sensor in the low temperature range and high temperature range are Equations (5) and (6), respectively. After the fitting was completed, we used the fitted Equation to compare with the actual test value. The nonlinear error was 0.07%, which was within the acceptable range, so the fitting was good.

(5) $$\begin{array}{c} y=11.01765-7.27157*10^{-5}*x \end{array}$$

(6) $$\begin{array}{c} y=11.19976-4.28571*10^{-4}*x \end{array}$$

In order to explore the sintering mechanism and failure reasons of the sensor, it was necessary to observe the microstructure of the same sensor under SEM in three stages after the screen printing, the sintering and the testing.

**Figure 11:**
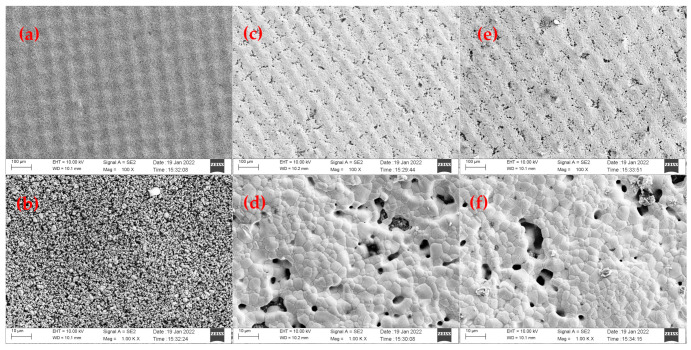
(**a**,**b**) Different magnifications of SEM images of sensor after screen printing, (**c**,**d**) different magnifications of SEM images of sensor after production, (**e**,**f**) different magnifications of SEM images of sensor after third testing.

The pictures obtained in SEM at different magnifications are shown in Figure 11. As shown in Figure 11a,b a regularly arranged silver paste film would be formed on the surface of the sensor with fine particles and uniform distribution when the screen printing was completed. During the sintering process, the organic components in the slurry gradually volatilized, leaving tiny pores. The grain size gradually increased, forming new grain boundaries, as shown in Figure 11c,d. When the sintering was completed, a dense layer of silver paste would be formed, and the film would have some holes. However, its area ratio was small. Its dense structure indicates that the sensor is durable and can work for a long time.

When the three tests were done, the sensor underwent long-term high-temperature operation with the surface porosity increasing. There were no changes in the crystal size of the film layer, though the larger silver crystals appeared, as shown in Figure 11e,f. The testing temperature was higher than the melting point of silver, which caused a small amount of silver on the surface to separate from the paste and recombine to form silver crystals.

If the working time continued to increase, the pores would gradually increase, and the number of silver crystals would add up until the film structure was destroyed and completely isolated particles, finally, the sensor would fail.

## 6. Conclusions

Two kinds of wireless passive high-temperature sensors and their corresponding broadband interrogation antennas were designed based on the “∗”-shaped structure, which was used in the alumina and silicon carbide ceramics as the base, respectively, in the study. By simulating a high-temperature environment of 1000 °C and repeating the experiment three times, the performance parameters of the two sensors were obtained. The testing results show that the sensor based on silicon carbide ceramics has a sensitivity of 73.68 kHz/°C at 25–500 °C and 440 kHz/°C at 500–1000 °C. The S11 value is from −30.316 dB to −16.184 dB, and its overall performance is better than the sensor based on alumina ceramic. Then, we explored the main reason for the failure of the sensor. The results of this paper suggest that the design of sensors based on silicon carbide ceramics has broad application prospects, and the use of slurry with a higher melting point helps to ensure long-term high-temperature stable operation of the sensor.

## Figures and Tables

**Table 1:** Sensor structure parameter table.

W1	L	S	θ	W2	D1	D2	Ø	H1
0.5	8.5	1.6	60°	15	1.5	2	1	1

**Table 2:** Inquiry antenna structure parameters.

W3	L3	A1	W4	L4	G	W5	L5	W6	L6	H2
30	92.5	8	3.6	4.5	0.5	1.43	80	9.25	**79.5**	**1**

**Table 3:** Comparison of various wireless passive temperature sensors in the literature.

Article	Material	Size	Sensitivity	Range	Ref
Xiong et al.	Alumina, Silver	16 × 8 × 2 mm^3^	0.19 MHz/°C	27–800 °C	[30]
Lin et al.	HTCC substrate and Platinum	103 × 25 mm^2^	0.279 MHz/°C	25–1100 °C	[31]
Idhaiam et al.	Al_2_O_3_, Tin-doped Indium Oxide	52 × 20.6 mm^2^	0.1 MHz/°C	500–1200 °C	[32]
Dan Yan et al.	AlN Ceramic, silver	22.4 × 34 × 1 mm^3^	104.77 KHz/°C	25–700 °C	[33]
This article	SiC, Silver	15 × 15 × 1 mm^3^	0.261 MHz/°C	25–1000 °C	
